# Changes in Browning Degree and Reducibility of Polyphenols during Autoxidation and Enzymatic Oxidation

**DOI:** 10.3390/antiox10111809

**Published:** 2021-11-15

**Authors:** Xuan Zhou, Aamir Iqbal, Jiaxing Li, Chang Liu, Ayesha Murtaza, Xiaoyun Xu, Siyi Pan, Wanfeng Hu

**Affiliations:** 1 College of Food Science and Technology, Huazhong Agricultural University, Wuhan 430070, China; zxquebec@webmail.hzau.edu.cn (X.Z.); aamirraoiqbal@webmail.hzau.edu.cn (A.I.); lijiaxing0220@webmail.hzau.edu.cn (J.L.); skliuc@webmail.hzau.edu.cn (C.L.); ayeshamurtaza@webmail.hzau.edu.cn (A.M.); xiaoyunxu88@gmail.com (X.X.); drpansiyi.hzau.edu@outlook.com (S.P.); 2 Key Laboratory of Environment Correlative Dietology, Huazhong Agricultural University, Ministry of Education, Wuhan 430070, China; 3 College of Agriculture and Life Sciences, Cornell University, Ithaca, NY 14853, USA

**Keywords:** polyphenol, autoxidation, enzymatic browning, eugenol, molecular docking

## Abstract

In the present study, the browning degree and reducing power of browning products of catechin (CT), epicatechin (EC), caffeic acid (CA), and chlorogenic acid (CGA) in autoxidation and enzymatic oxidation were investigated. Influencing factors were considered, such as pH, substrate species and composition, and eugenol. Results show that polyphenols’ autoxidation was intensified in an alkaline environment, but the reducing power was not improved. Products of enzymatic oxidation at a neutral pH have higher reducing power than autoxidation. In enzymatic oxidation, the browning degree of mixed substrates was higher than that of a single polyphenol. The reducing power of flavonoid mixed solution (CT and EC) was higher than those of phenolic acids’ (CA and CGA) in autoxidation and enzymatic oxidation. Eugenol activity studies have shown that eugenol could increase autoxidation browning but inhibit enzymatic browning. Activity test and molecular docking results show that eugenol could inhibit tyrosinase.

## 1. Introduction

Polyphenols are widely found in plants [1], including flavonoids and phenolic acids [2]. Polyphenols are secondary metabolites, which play an important role in the browning of some fruit and vegetables. The direct contact between polyphenols and oxygen produces colored polymers and unpleasant flavors that affect the color and other sensory qualities of processed products [3].

Browning of polyphenols includes enzymatic and non-enzymatic browning. Enzymatic browning is catalyzed by polyphenol oxidase (PPO), which can catalyze the reaction of hydroxylation of monophenols to *o*-diphenols (cresolase activity) [4]. PPO can also catalyze the oxidation of *o*-diphenols to the corresponding *o*-quinones (catecholase activity) [5]. Mushroom tyrosinase is a type of PPO that can catalyze two distinct types of reactions: hydroxylation of monophenols to o-catechol and oxidation of catechol to o-quinone [6]. Quinones are polymerized and generate polymeric compounds through different reaction pathways (such as coupling reactions) [7]. In general, enzymatic browning is faster than non-enzymatic and involves more complex oxidative processes and products.

**Scheme 1:**
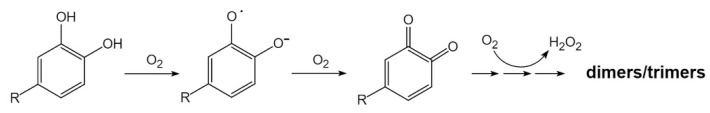
Mechanism of autoxidation of o-diphenols.

In the absence of enzymes, phenolic compounds can also undergo autoxidation process and produce browning products [8]. The reaction happens more quickly in alkaline conditions [9]. This non-enzymatic autoxidation of o-diphenols occurs as follows. The first step is the oxidation of *o*-diphenols molecules to semiquinone intermediates and superoxide anions [10]. The second step is the subsequent oxidation of semiquinone intermediates with oxygen to generate quinones [11], whose reaction rate is slower than two-electron (the first step) oxidation. Thus, the latter is the rate-determining step. Quinones can polymerize with polyphenol monomers to form polymers, such as dimers and trimers (Scheme 1) [12].

Reducing power is an important parameter related to antioxidant properties. Most polyphenols have good reducibility [13], which could hinder the further oxidation of other food components. The phenolic hydroxyl groups at the ortho position can scavenge free radicals, breaking the free radical chain reaction [14]. However, previous studies have shown that the oxidation products still have high reducibility [15]. During the processing of black tea, catechin (CT) undergoes oxidation and polymerization [16]. Researchers [17] found that the radical-scavenging activities of black tea were not reduced. The oxidation products of epicatechin (EC) could exert an inhibitory effect on enzymatic browning [18]. These findings suggest that oxidation products could lessen browning and may have reducibility.

At present, there are few studies on the correlation between browning degree and antioxidant activity. External and internal factors affect the browning degree and reducing power of phenolic compounds during oxidation. pH value has a great impact on the structure of polyphenol substrates. The concentration of the substrate may affect the PPO, thereby affecting the browning degree. There are synergistic and antagonistic interactions between flavonoids that may affect browning [19]. Eugenol is one phenolic compound extracted from clove essential oil that can scavenge radicals, which reduces the oxidation of polyphenol [20].

This study simulated oxidation systems, including enzymatic and autoxidation. We investigated the effects of external factors such as pH adjustment and inhibitor eugenol and internal factors such as substrate species and composition (CT, EC, Caffeic acid (CA), Chlorogenic acid (CGA), and mixed substrates) on the browning degree and reducing power with time. Moreover, molecular docking was performed to further investigate the binding of polyphenols and eugenol to enzymes. It further explained why the browning degree of four samples was different under the same simulation conditions and the mechanism of eugenol inhibiting browning. We tried to find the relationship between browning degree and reducing power under various conditions so as to achieve browning products with moderate browning and good antioxidant properties.

## 2. Materials and Methods

### 2.1. Chemicals

Standard polyphenol compounds, including CT(CAS: 154-23-4, BR), CGA(CAS: 327-97-9, BR), EC (CAS:490-46-0, HPLC), CA (CAS: 331-39-5, AR), and eugenol, were purchased from Shanghai Yuan Bio-Technology Co., Ltd. (Shangai, China). Other analytical grade reagents were provided by sinopharm chemical reagent Co., Ltd. (Shanghai, China). Polyphenol oxidase (mushroom tyrosinase, EC 1.14.18.1, 1000 U/g) was obtained from Sigma-Aldrich Co. (Saint Louis, MO, USA)

### 2.2. Oxidation Simulation System

#### 2.2.1. Autoxidation System

Disodium hydrogen phosphate-citric acid buffer was prepared with disodium hydrogen phosphate, sodium dihydrogen phosphate, and citric acid and adjusted to the desired pH value by hydrochloric acid and sodium hydroxide. A 1 mmol/L solution of polyphenol (CT, EC, CA, and CGA) was incubated in disodium hydrogen phosphate-citric acid buffer at pH 3.5. The reaction was carried out at room temperature in 19.5 mL of the solution. Samples were collected after 0, 1, 2, 3, 5, and 7 days of storage.

#### 2.2.2. Enzymatic Oxidation System

A 2 mmol/L solution of polyphenol (CT, EC, CA, and CGA) was incubated in disodium hydrogen phosphate-citric acid buffer at pH 6.8. An aliquot of 50 mg/L Polyphenol oxidase was added, and the solution was incubated at room temperature. Samples were collected after 0, 5, 10, 20, and 30 min of storage.

#### 2.2.3. Preparation of Eugenol Emulsion

Based on the previous study [21], the preparation of eugenol was as follows: Span-80 (1 g) and Tween-80 (19 g) were mixed with eugenol (2 g) in distilled water to the final concentrations of 0.5, 1.0, and 2.0% (*m*/*v*). 1 mL 0.5%, 1.0%, and 2.0% (*m*/*v*) eugenol emulsion were dissolved in the 19.5 mL substrate solution, respectively. Samples collection was consistent with Section 2.2.1 and Section 2.2.2.

### 2.3. Browning Degree Analysis

The browning degrees of the four polyphenol solutions were estimated using a Multiskan GO Spectrum (Thermo Scientific, Waltham, MA, USA) [22]. The absorbance of the samples was measured at 420 nm.

### 2.4. Reducing Power Analysis

A 1.0 mL of sample was mixed with 0.5 mL of sodium phosphate buffer (0.2 mol/L, pH = 6.6) and 0.5 mL of 1% (*m*/*v*) potassium ferrocyanide and then incubated in a water bath at 50 °C for 20 min. Then, 0.5 mL of 10% (*m*/*v*) trichloroacetic acid was added to the mixture and centrifuged. The supernatant (0.5 mL) was mixed with 0.5 mL distilled water and 0.1 mL of 0.1% (*m*/*v*) ferric chloride solution. After 10 min, the absorbance was obtained at 700 nm [23].

### 2.5. Molecular Docking

Docking was performed using Autodock Vina to identify binding poses of the four compounds within the active center of mushroom tyrosinase. The crystal structure of mushroom tyrosinase (PDB ID: 2Y9X) was obtained from Protein Data Bank (http://www.rcsb.org/pdb accessed on 28 June 2020). As the crystal structure of mushroom tyrosinase consists of four similar parts with similar active sites, thus chain A was chosen for molecular docking. The three-dimensional structure of four substrates (CT, EC, CA, and CGA) and eugenol were downloaded on Open Chemistry Database (PubChem, http://pubchem.ncbi.nlm.nih.gov/ accessed on 28 June 2020). The Auto Dock Tools 1.5.6 package was employed to generate the docking input files. The search grid of the key site of tyrosinase was identified as center x: −10.0, center y: −28.7, and center z: −43.4 with dimensions size x: 10.9, size y: 13.7, and size z: 14.3. The Lamarckian genetic algorithm was used for conformational sampling of the compounds. The best binding pose of the complex was the model with the lowest binding energy. The molecular docking results were visualized by programs PyMOL (http://www.pymol.org/ accessed on 2 July 2020) and LigPlot+ (https://www.ebi.ac.uk/ accessed on 2 July 2020).

### 2.6. Statistical Analysis

The results are presented as the mean ± standard deviation of three replicates, performed on a number of samples for each experiment. One-way ANOVA was used to compare the means, and the least significant difference test showed the values were statistically different. Differences were considered significant at *p* < 0.05.

## 3. Results

### 3.1. Oxidation of Four Polyphenols at Different pH Values

#### 3.1.1. Autoxidation

**Figure 1:**
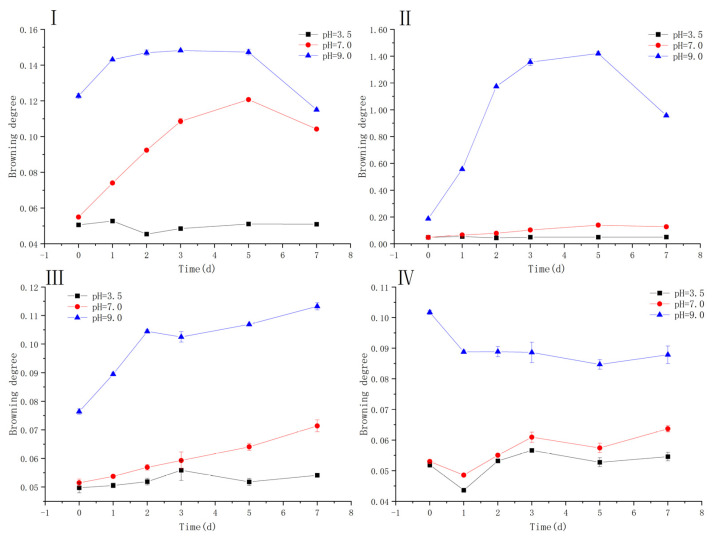
The browning degree of autoxidation products of (**I**) Catechin (**II**) Epicatechin (**III**) Caffeic acid (**IV**) Chlorogenic acid in various values of pH.

Browning of polyphenols can reflect the degree of oxidation of phenols to some extent [24]. pH value could greatly affect the colors of polyphenols, which could be demonstrated by browning degree [25]. The products of these polyphenols showed regular changes in browning degree under different pH conditions (Figure 1).

The browning degree of four samples (CT, EC, CA, and CGA) significantly increased with increasing pH and prolonged reaction time, compared with that at day 0 (*p* < 0.05). At pH 3.5, the browning degree of the four samples in the simulation systems did not change significantly within 7 days (Figure 1). The acidic environment inhibited autoxidation [9]. However, the browning degree of the four solutions increased at pH 9.0. This result was consistent with a previous finding that green tea polyphenols undergo self-polymerization under weak alkaline conditions [26]. It is reported that a higher pH value is closely related to higher accumulation of the superoxide anion radicals and semiquinone intermediates, and then more semiquinones would be oxidized to *o*-quinones, which would lead to the formation of brown polymers [10]. On the other hand, the absorbance values at 420 nm of the samples of CT and EC decreased on day 5, which may be due to the rapid oxidation of CT and EC at high pH in autoxidation system. In the later stages of oxidation, the oxidation products may continue to polymerize, causing the absorption peak of the sample to deviate from 420 nm [12].

**Figure 2:**
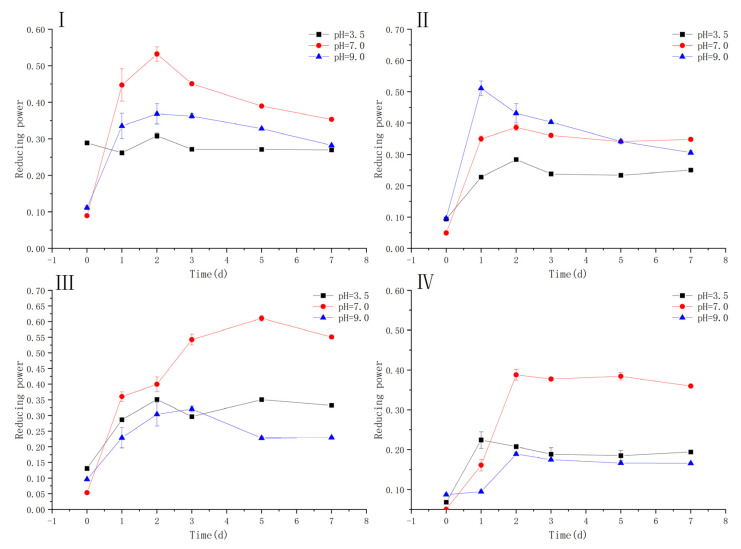
The Reducing power of autoxidation products of (**I**) Catechin (**II**) Epicatechin (**III**) Caffeic acid (**IV**) Chlorogenic acid in various values of pH.

Reducing power is an important index to measure antioxidant activity [27]. The greater the value, the stronger the reducing force was. Based on the experimental data (Table 1 and Figure 2), the change in reducing power could be divided into two stages by time. In the first stage, the reducing power quickly increased, especially for CT, EC, and CA. In the second stage, the reducing power stabilized with time in the acidic environment, and the change was not significant for all samples. Except for EC, the reducing powers of CT, CA, and CGA were the maximum at pH 7.0. At pH values of 3 and 9, the reducing power was low. Taking CA as an example, the maximum reducing power at pH 3 and 9 was 42% and 47%lower than that at pH 7, respectively. When the pH value was high, the browning degree of phenolic substances increased, indicating that the oxidation degree was too high, so the reducing power was low. It is noteworthy that at lower pH values, phenolic substances almost did not undergo oxidative browning, but their reducing power was still far lower than that of moderately browning substances. Therefore, it is speculated that moderate browning at a neutral pH could achieve higher reducing power.

#### 3.1.2. Enzymatic Oxidation

The enzymatic browning of substrates was investigated in acidic and neutral environments to avoid the interference of autoxidation in the alkaline environment.

**Figure 3:**
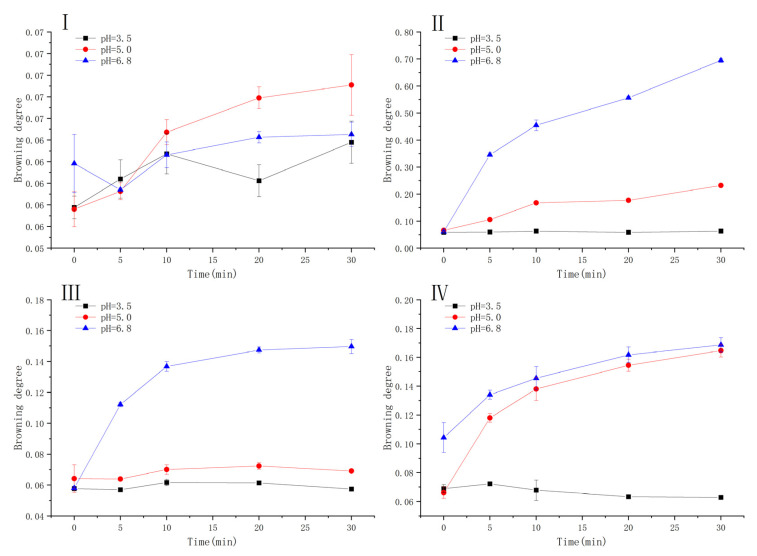
The browning degree of enzymatic browning products of (**I**) Catechin (**II**) Epicatechin (**III**) Caffeic acid (**IV**) Chlorogenic acid in various pH values.

As shown in Figure 3, there was no significant difference in the browning degree of CT under different pH values after enzymatic oxidation (*p* > 0.05), and all of the browning degrees were less than 0.07. Under the acidic condition, the browning degree of the four samples was less than 0.1, which did not change significantly with time and showed few colored polymers. The optimum pH of mushroom tyrosinase was 6.8, and the browning degree of the other products increased significantly with time except for CT (*p* < 0.05). In addition, the browning degrees of CA and CGA were lower than that of EC. This result indicated that EC was more prone to browning than CA and CGA. This result may be attributed to the 3-R configuration at carbon-3 of EC, with less steric hindrance, faster kinetic speed, and easier polymerization into dimer compared with CA and CGA [28]. As mentioned in Section 3.1.1, the acidic environment restrained the oxidative polymerization of CT, EC, CA, and CGA. Similarly, low pH values reduce the formation of colored polymers in enzymatic oxidation. Colorless polymers are produced at low pH values, whereas yellow compounds are produced at high pH values [29,30]. Acidic environments could also change the conformation of enzymes and disrupt some of the stable intermolecular forces [31], such as hydrogen bonding and hydrophobic interactions, resulting in activity loss.

**Figure 4:**
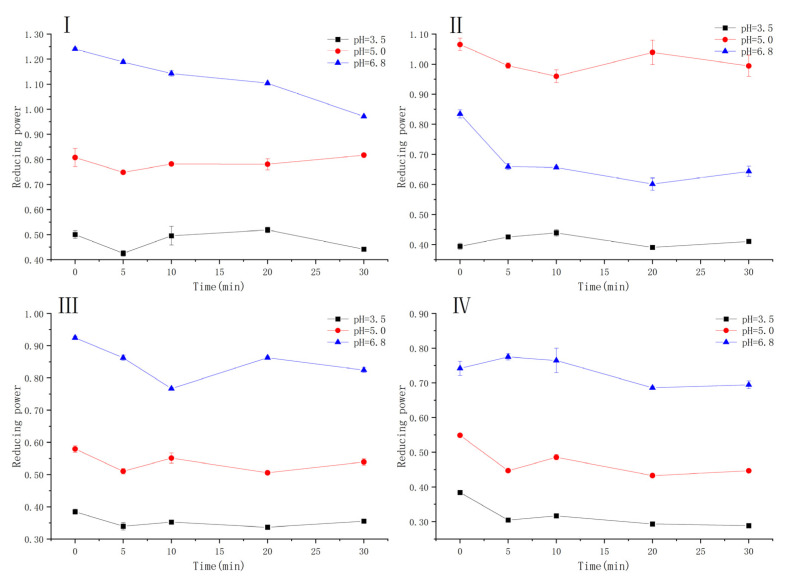
The reducing power of enzymatic browning products of (**I**) Catechin (**II**) Epicatechin (**III**) Caffeic acid (**IV**) Chlorogenic acid in various values of pH.

In the enzymatic oxidation, the reducing power showed a significant difference at different pH values (*p* < 0.05). Under pH 3.5, the reducing power of the polyphenols was all less than 0.4 and lower than that under the two other conditions (Figure 4). At neutral pH value, the reducing power on day 7 was 120%, 56.6%, 132%, and 141% higher than that in acidic environments, respectively. Similar to the reducing power of autoxidation samples, polyphenols that oxidized to a moderate level at neutral pH in the presence of the enzyme would have high reducing power. At pH 6.8, the reducing power of the products of enzymatic browning increased to as much as 1.0 for CT after 30 min of reaction. Therefore, reducing power of polyphenols at neutral pH in the presence of enzyme was much higher than those of the autoxidized ones. This result suggested that enzymatic browning has positive effects to improve antioxidant activity when appropriately utilized.

### 3.2. Oxidation of Four Polyphenols at Different Concentration

#### 3.2.1. Autoxidation

Due to the acidic environment, the browning degree of all autoxidation samples at different concentrations was less than 0.058 for 7 days (data not shown).

**Figure 5:**
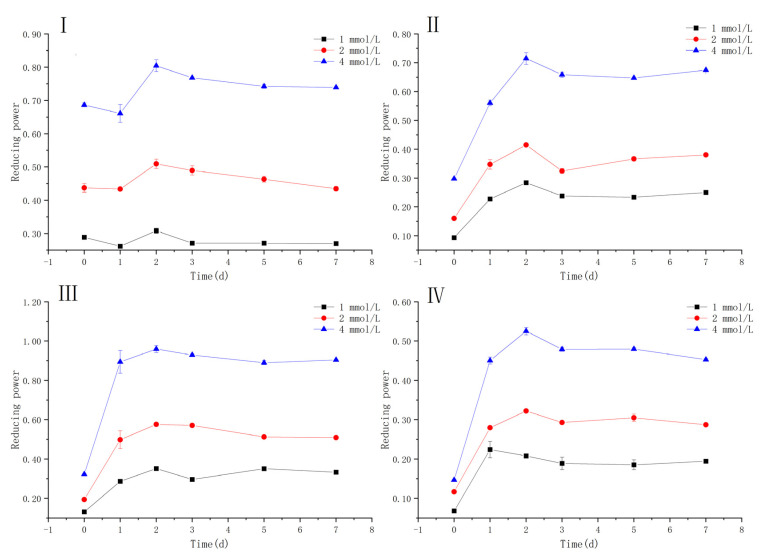
The reducing power of autoxidation products of (**I**) Catechin (**II**) Epicatechin (**III**) Caffeic acid (**IV**) Chlorogenic acid in various concentrations at pH 3.5.

However, the reducing power of each substrate sample at various concentrations was significantly different. As shown in Figure 5, the higher the concentration of substrate, the higher the reducing power of products and the faster the increasing rate of reducing power in the first 2 days. As the reaction time was prolonged, the reducing power remained steady for the following days. Taking CT as an example, when the substrate concentration was 2 and 4 mmol/L, its reducing power on day 7 was 1.6 times and 2.7 times that of 1 mmol/L, respectively. This result suggested that the reducing power of the solutions was concentration-dependent.

#### 3.2.2. Enzymatic Oxidation

Under enzymatic oxidation, the solutions with different substrate concentrations showed a significant difference in browning degree (*p* < 0.05).

**Figure 6:**
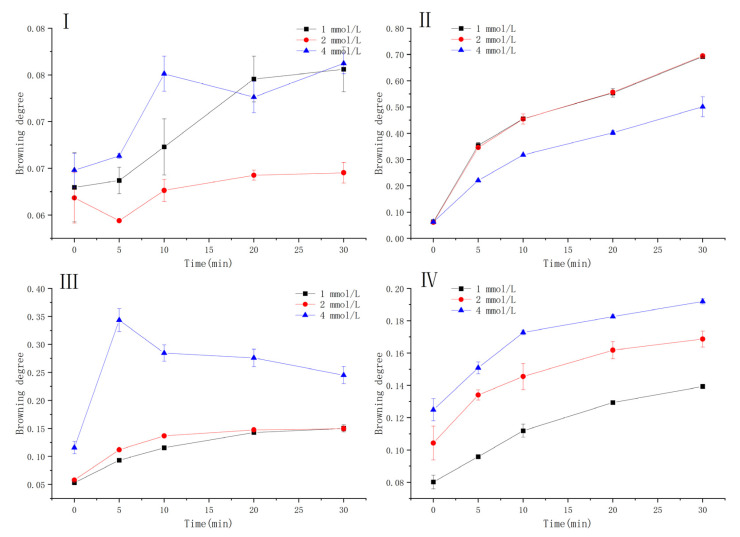
The browning degree of enzymatic browning products of (**I**) Catechin (**II**) Epicatechin (**III**) Caffeic acid (**IV**) Chlorogenic acid in various concentrations at pH 6.8.

As shown in Figure 6, for phenolic acids (CA and CGA), the browning degree and its reaction rate increased with increasing concentration. The browning degree of 4 mmol/L CA was 3 times as much as 1 mmol/L CA at 5 min. More substrates may be in contact with the enzyme to produce more browning products. In a previous study, high substrate concentration readily led to the generation of colored compounds [25]. However, the flavonoids (CT and EC) did not conform to this rule. The browning degree of the 4 mmol/L EC solution was lower than those of the two other concentration groups. This result may be attributed to the high substrate concentrations, which obtained more products and inhibited tyrosinase [18]. Moreover, the distinction between CT and EC suggested that the molecular structure of the phenols affected enzymatic oxidation and subsequent coupling reaction. In acidic conditions, most of the initial products did not contribute to browning [12], especially CT, CA, and CGA. Therefore, their browning degrees were smaller than those of EC.

**Figure 7:**
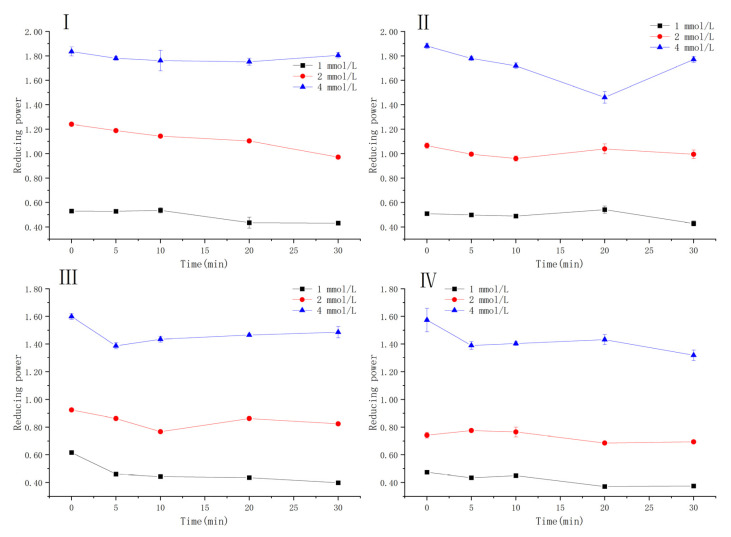
The reducing power of enzymatic browning products of (**I**) Catechin (**II**) Epicatechin (**III**) Caffeic acid (**IV**) Chlorogenic acid in various concentrations at pH 6.8.

The reducing power of the solutions positively correlated with the substrate concentration (*p* < 0.05). As shown in Figure 7, the reducing power of each substrate sample at various concentrations was significantly different in the enzymatic oxidation. At 30 min, 4 mmol/L CT was about 3 times the reducing power of 1 mmol/L CT, and 2 mmol/L CT was about 2 times the value of 1 mmol/L CT. This result may be because polyphenol is more prone to browning under the catalysis of PPO, and moderate browning products had good reducibility [18,32]. This result is consistent with the finding of a previous study that CT and theaflavins have strong antioxidative properties because the dimers have many hydroxyl groups that can scavenge free radicals [33].

### 3.3. Oxidation of Mixed Substrates

**Figure 8:**
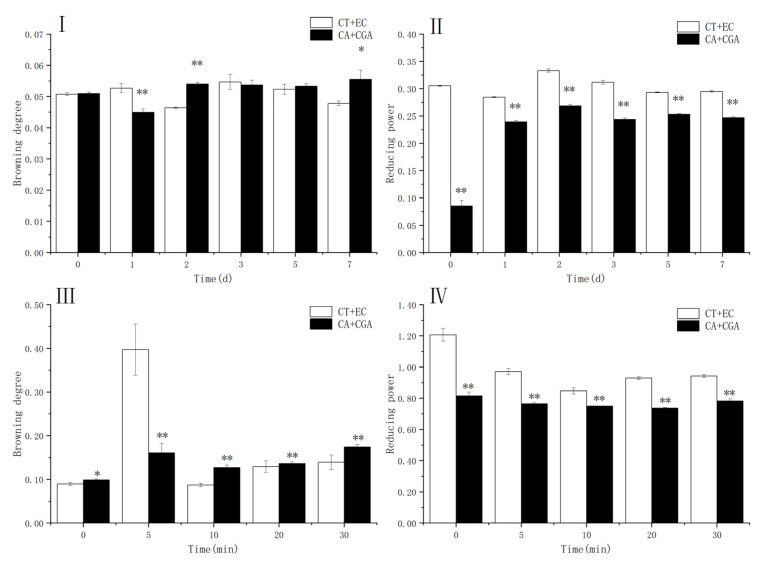
The browning degree and reducing power of (**I, II**) autoxidation and (**III, IV**) enzymatic browning products of flavonoid (Catechin + Epicatechin) and phenolic acid (Caffeic acid + Chlorogenic acid). * Means *p* < 0.05 significant correlation level; ** Means *p* < 0.01 more significant correlation level.

There was no significant difference in the browning degree of flavonoid mixed solution (CT and EC) and phenolic acid mixed solution (CA and CGA, except for days 1, 2, and 7) for the autoxidation samples (Figure 8I). In terms of enzymatic browning (Figure 8III,IV), the browning degree of the flavonoid mixed solution (CT and EC) fluctuated between 0.09 and 0.14 (except for the value at 5 min) and phenolic acids between 0.10 and 0.17. However, the reducing power of the flavonoids (CT and EC) decreased from 1.20 to 0.94, while the value remained at 0.80 in the enzymatic oxidation. The reducing power of the phenolic acids is lower than that of the flavonoids in both autoxidation and enzymatic oxidation (Figure 8II,IV).

According to free radical theory, the C-ring of the two flavonoid molecules could donate electrons to the ortho-dihydroxy structure in the B-ring, making the oxyhydrogen bond of catechol easy to break [19]. Then free radicals can easily combine with superoxide anions or other free radicals to generate stable structures and break the chain reaction of oxidation [14]. Therefore, it restrained the browning reaction.

Different from flavonoids (CT and EC), CA and CGA are simple phenolic acids. The para-hydroxyl groups of CA and CGA are electron-withdrawing groups, and their oxyhydrogen bonds are relatively stable [34]. However, the phenolic hydroxyl group of flavonoids (CT and EC) easily undergoes two-electron oxidation, leading to better reducibility, while the browning degree of flavonoids (CT and EC) was higher than that of phenolic acids (CA and CGA).

**Figure 9:**
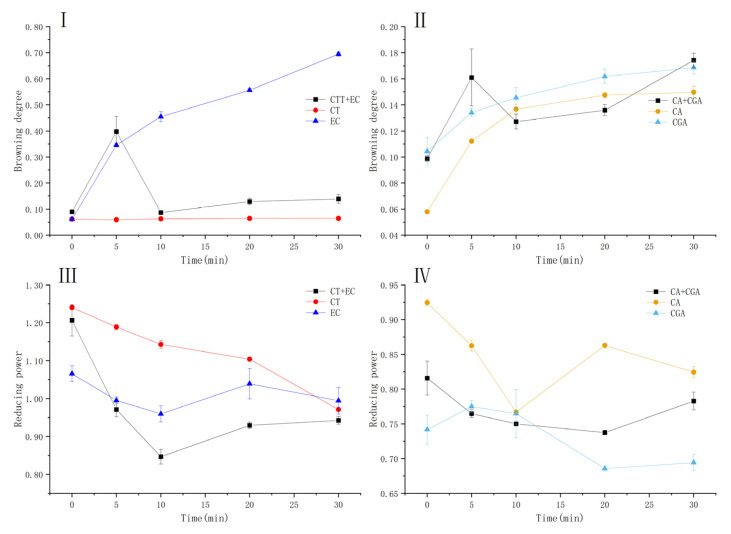
The browning degree and reducing power of enzymatic browning products in various combined substrates (**I,III**) Catechin + Epicatechin (**II**,**IV**) Caffeic acid + Chlorogenic acid.

In the enzymatic oxidation, the browning degree of EC reached the maximum 0.69. The browning degree of CT and flavonoid mixed solution (CT and EC) remained 0.08 and 0.13, respectively (Figure 9). Under pH 6.8, the browning of EC was more severe than other samples. Similarly, the browning degree of CGA was 0.16, higher than that of CA (0.15) and that of the mixed solution (0.14, CA and CGA).

For the flavonoid mixed solution (CT and EC), the reducing power was between CT and EC at the beginning of the experiment. As the reaction progressed, the parameter of the mixed solution (CT and EC) was lower than that of two separate solutions. Previous studies have found that CT and EC exert antagonistic effects on scavenging free radicals [19], which reduced the reducing power of the mixed solution. At the end of the reaction (30 min), the reducing power of CA, mixed solution (CA and CGA), and CGA were 0.82, 0.78, and 0.69, respectively. The enzymatic products of CA have the best reducibility in phenolic acid samples. A previous study indicated that CA and ferulic acid act antagonistically in DPPH and ABTS assays [35].

### 3.4. Oxidation of Four Polyphenols with Eugenol

#### 3.4.1. Autoxidation

**Figure 10:**
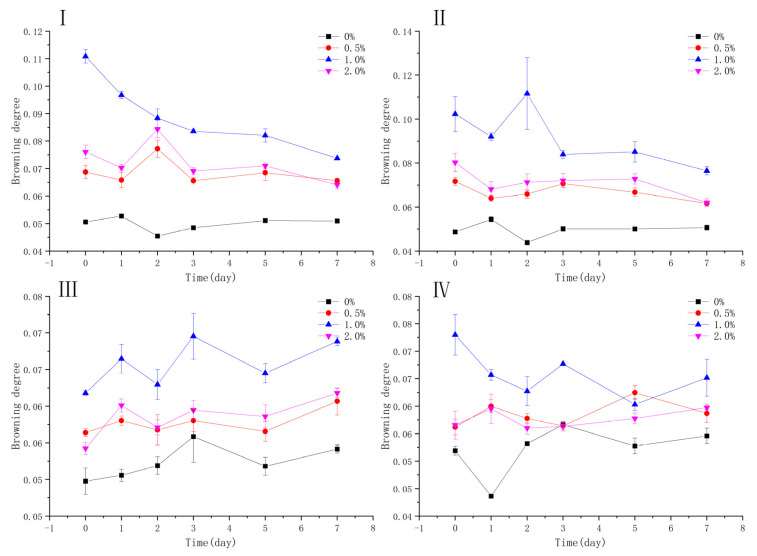
The browning degree of autoxidation browning products of (**I**) Catechin (**II**) Epicatechin (**III**) Caffeic acid (**IV**) Chlorogenic acid under the effect of different concentrations of eugenol.

The eugenol treatment significantly increased the browning degree of four samples in the autoxidation system (*p* < 0.05). As shown in Figure 10, at 1% eugenol concentration, the browning degree of four samples was the highest, with the maximum reaching 0.11, 0.11, 0.07, and 0.07. However, when the concentration of eugenol was 2%, the browning degree of samples decreased to 0.06 on day 7. One possible explanation for this result is that eugenol could be converted into semiquinone radicals as a phenolic compound [36]. Moreover, the semiquinone radical and superoxide anion played a catalytic role in autoxidation [11]. Still, the increase in semiquinone radicals promoted the conversion of more catechol into polymers, which raised the browning degree.

**Figure 11:**
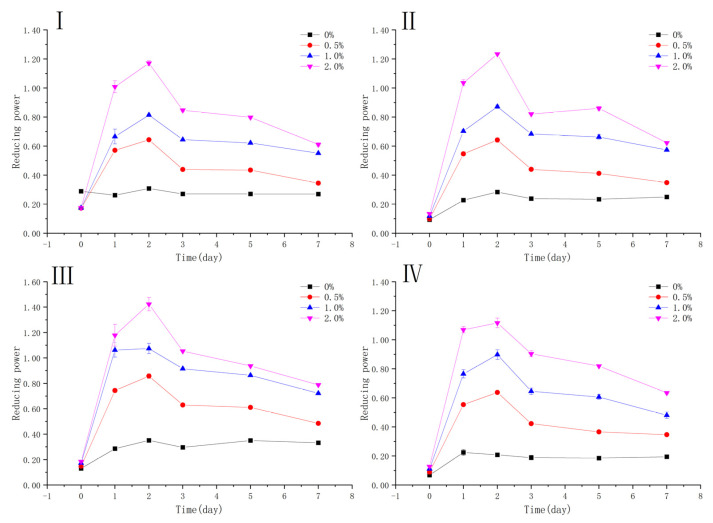
The reducing power of autoxidation browning products of (**I**) Catechin (**II**) Epicatechin (**III**) Caffeic acid (**IV**) Chlorogenic acid under the effect of different concentrations of eugenol.

With the increasing eugenol concentration, the reducing power of autoxidation products increased. The reducing power of the solution with 2% eugenol rose sharply to 1.2, then decreased slowly, and finally stabilized at 0.6–0.8. Still, it was always higher than that of the solution without eugenol added (Figure 11). This result may be because eugenol is an antioxidant with good reducibility [37]. Moreover, it further demonstrated that the browning degree is not negatively correlated with the reducing capability.

#### 3.4.2. Enzymatic Oxidation

**Figure 12:**
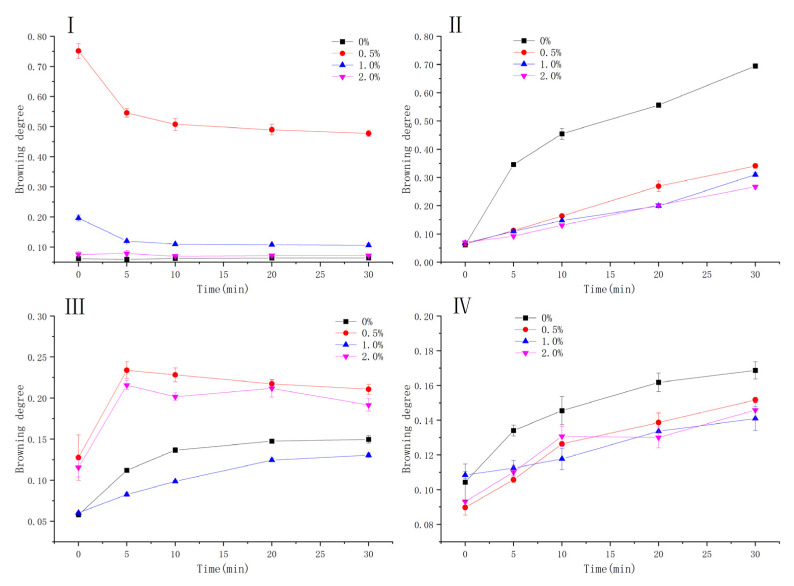
The browning degree of enzymatic browning products of (**I**) Catechin (**II**) Epicatechin (**III**) Caffeic acid (**IV**) Chlorogenic acid under the effect of different concentrations of eugenol.

For EC and CGA solutions, the enzymatic browning degree decreased by increasing eugenol concentrations, and the eugenol treatment could effectively inhibit the enzymatic browning. However, the browning degree of CT with eugenol concentrations of 0.5% and 1% was higher than that of the control group. The browning degree of CA with 0.5% and 2.0% eugenol concentrations was 41% and 28% higher than that of the control group (Figure 12). For CA and CGA substrates, 1% eugenol had the best inhibition effect on the browning of polyphenol. Therefore, the 1% concentration of eugenol was effective for inhibiting the enzymatic browning. It may be because the ability of 2% eugenol to promote autoxidation is stronger than its ability to inhibit PPO, thus increasing the degree of browning of polyphenols.

**Figure 13:**
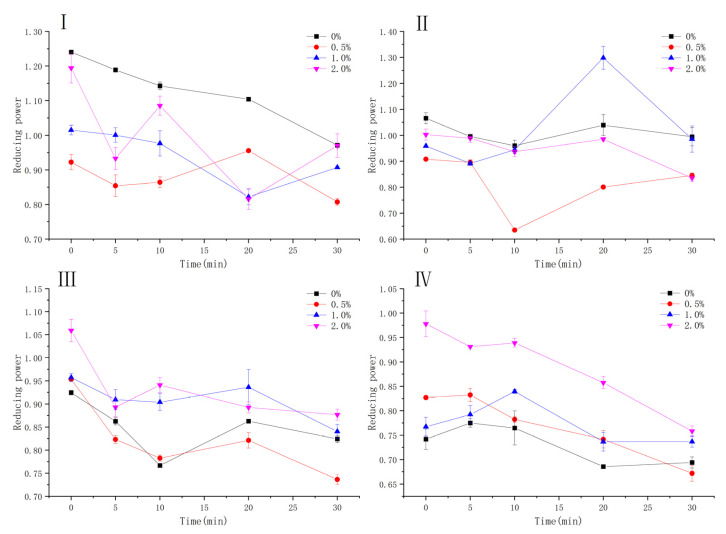
The reducing power of enzymatic browning products of (**I**) Catechin (**II**) Epicatechin (**III**) Caffeic acid (**IV**) Chlorogenic acid under the effect of different concentrations of eugenol.

As shown in Figure 13, the reducing power of the control group of CT was 1.24 at 0 min. The value of CT with 0.5% eugenol was 0.92, and that CT with 1% eugenol was 1.02. The reducing power of CT containing 2% eugenol fluctuated between 0.82 and 1.20. The eugenol reduced the reducing power of browning products for CT and EC. It is noteworthy that the reduction effect of high concentration (2.0%) of eugenol was less than that of low concentration (0.5%).

The reducing power of the control group of CGA was 0.74 compared to 0.84 and 0.77 of 0.5% eugenol and 1.0% eugenol at 0 min. The value of CT with 2% eugenol was 0.98. For CA and CGA, eugenol increased the reducing power of enzymatic products. Moreover, the higher the concentration, the greater the increase. Eugenol could scavenge radicals [38]. Therefore, a large amount of eugenol raised the reducing power of the products of CA and CGA.

### 3.5. Computational Docking Simulation of Polyphenols Binding to Tyrosinase

**Figure 14:**
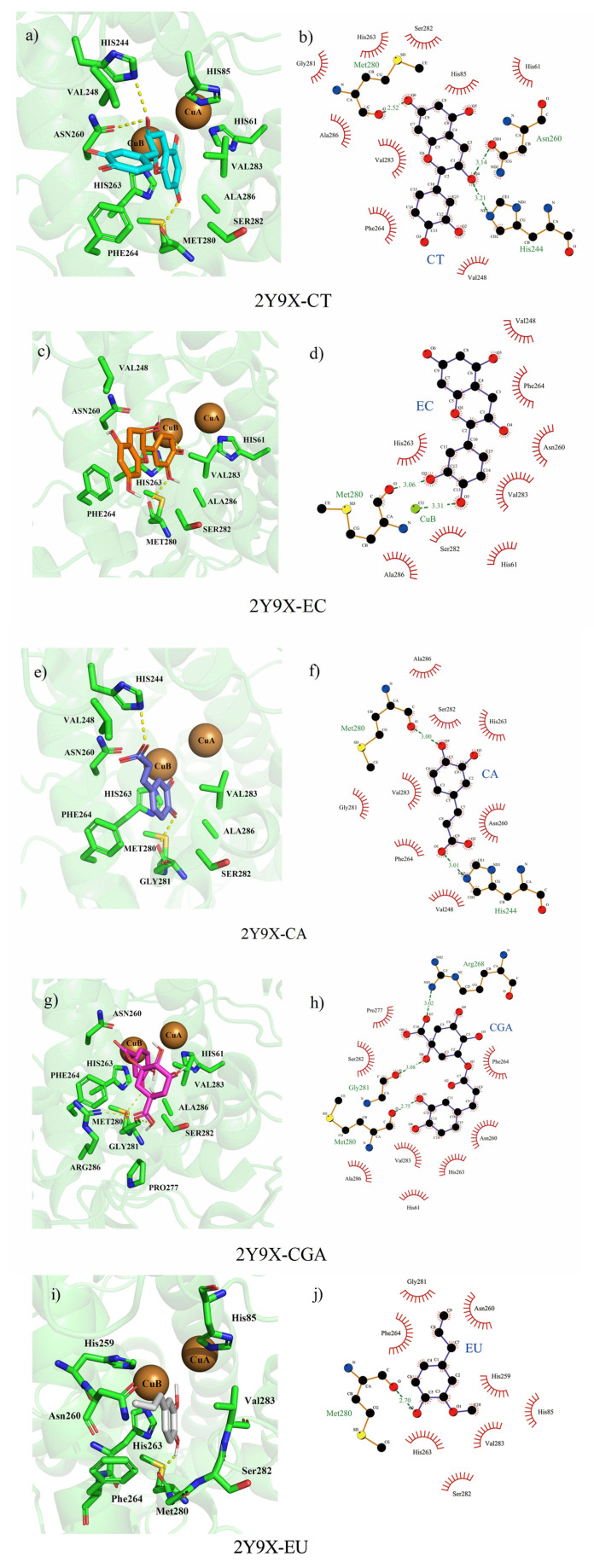
Molecular docking conformations of five compounds and tyrosinase. The image on the left is a 3D visual image, and the image on the right is a 2D visual image. (**a**,**b**) Detailed view of the binding mode between the catechin and the tyrosinase (**c**,**d**) Detailed view of the binding mode between the epicatechin and the tyrosinase (**e**,**f**) Detailed view of the binding mode between the caffeic acid and the tyrosinase (**g**,**h**) Detailed view of the binding mode between the chlorogenic acid and the tyrosinase (**i**,**j**) Detailed view of the binding mode between the eugenol and the tyrosinase.

The docking conformations of the tyrosinase and four compounds (CT, EC, CA, and CGA) are shown in Figure 14. The CT, EC, CA, and CGA were docked into the key active sites of the tyrosinase, respectively.

2Y9X-CT: Conformation of the interaction between CT with tyrosinase. 2Y9X-EC: Conformation of the interaction between EC with tyrosinase. 2Y9X-CA: Conformation of the interaction between CA with tyrosinase. 2Y9X-CGA: Conformation of the interaction between CGA with tyrosinase. 2Y9X-EU: Conformation of the interaction between eugenol with tyrosinase.

Table 2 described the detailed analysis of computational docking. It included the affinity of four ligands (CT, EC, CA, and CGA) and receptors (tyrosinase), amino acid residues involved in the formation of hydrophobic interactions and in the formation of hydrogen bonds. The lower the binding affinity value, the stronger the affinity between tyrosinase and substrate will be [39]. The experimental data of browning degree showed that the enzymatic browning of all CT groups (except those with eugenol addition) was not obvious. Therefore, we hypothesized that CT was not the tyrosinase substrate in our experiment. However, the lowest binding affinity was predicted to be −7.0 kcal/mol for CT, −6.7 kcal/mol for EC, −6.3 kcal/mol for CA, and −6.9 kcal/mol for CGA. CT’s affinity is the lowest among the four compounds, indicating that CT binds were very strongly linked to tyrosinase. The value of this affinity between tyrosinase and CT does not negate the hypothesis that CT is not the substrate of tyrosinase. Tyrosinase catalyzes the oxidation of ortho-phenol but cannot catalyze the oxidation of meta-phenol and para-phenol [12]. At optimal conformation, the resorcinol groups of CT extend into the active center with the lowest binding energy, which means CT cannot serve as substrates for mushroom tyrosinase. While the catechol groups of the other three compounds (EC, CA, and CGA) can extend into the active center, indicating that they can serve as substrates. Further analysis showed that in addition to hydrophobic interactions and hydrogen bonding, π-π stacking interaction between His263 or/and Phe264 and substrates (CT, EC, CA, and CGA) could also stabilize the binding of tyrosinase with the four ligands.

## 4. Discussion

The pH, substrate species, concentration, and eugenol affect phenolic compounds’ autoxidation and enzymatic oxidation. The acidic conditions (pH 3.5) can reduce autoxidation and enzymatic browning by inhibiting the initial oxidation. In contrast, the alkaline condition allowed the browning degree to reach the highest for all samples. As concerned about the reducing power, autoxidation and enzymatic oxidation behaved differently. For autoxidation, the reducing power of EC, CA, and CGA under acidic and alkaline conditions (pH 9.0) were lower than those under neutral conditions (pH 7.0). Nevertheless, the browning degree of polyphenol compound in alkaline conditions was higher than that in acidic conditions. This result suggested that autoxidation of polyphenols in alkaline environment was intensified, but the reducing power was not improved. In enzymatic oxidation, the reducing power increased with increasing pH value (pH 3.5 to pH 6.8). Polyphenols that oxidized to a moderate level at neutral pH would have high reducing power in the presence of the enzyme. It indicated that enzymatic oxidation of polyphenols was beneficial to the improvement of reducing power.

The browning degree increased with increasing substrate concentration for CA and CGA under enzymatic conditions. It showed that the degree of enzymatic oxidation is closely related to the substrate concentration. Moreover, both autoxidation and enzymatic oxidation may cause an increase in the reducing power of all samples with the growth of substrate concentration. The reducing power increased with the increasing substrate concentration, whether the polyphenols were oxidized or not. High polyphenol concentration could effectively provide high reducing power.

The changes in the browning degree of autoxidation were not significant for the mixed substrates. Mixed polyphenols do not promote autoxidation reactions. Compared with mixed substrates, the enzymatic browning of single polyphenols was more severe. Regarding the reducing power, flavonoid mixed solutions (CT and EC) had higher reducing power than that of phenolic acid mixed solution (CA and CGA) for autoxidation and enzymatic browning. The reducing power of flavonoid mixed solution (CT and EC) was lower than that of the single polyphenol in enzymatic browning. CT and EC may exert antagonistic effects on enzymatic oxidation.

The eugenol treatment may enhance the browning degree of four samples in autoxidation. When the eugenol concentration was 1.0%, the value of browning degree reached the maximum. Moreover, the eugenol treatment also increased the reducing power of solutions in the autoxidation browning. It indicated that eugenol-induced autoxidation could improve the reducing power of the reaction system. In the enzymatic oxidation, eugenol could inhibit the browning reaction. The molecular docking results further analyzed the interaction between ligands and enzymes. CT was not the substrate for mushroom tyrosinase, while the other three compounds (EC, CA, and CGA) could serve as substrates. Activity test and molecular docking results suggested that eugenol could inhibit PPO by binding to its active site.

## 5. Conclusions

In this work, we investigated the effects of pH, substrate species and composition (CT, EC, CA, CGA, and mixed substrates) and eugenol on the browning degree and reducing power with time. Based on the above results, we speculate that the reducing power, degree of browning, and degree of polymerization are closely related. The autoxidation of polyphenol often generates quinones, which are lighter in color. Nevertheless, enzymatic oxidation led to the generation of the conjugate structures, making the color of oxidized products deeper [40]. Besides, the colored polymers produced by oxidative coupling in enzymatic oxidation have more phenolic hydroxyl groups than in autoxidation [41]. Therefore, its reducing power of enzymatic oxidation product is higher than that of autoxidation under the same conditions. It provides a new idea for producing products with moderate browning and good antioxidant properties.

## Figures and Tables

**Table 1:** Reducing power (Absorbance Value at 700 nm) of autoxidation products (different pH).

	Absorbance Value at Day 0	Maximum	Maximum Corresponding Time/Day	Largest Increase Rate	Increase Rate at Day 7
CT					
pH = 3.5	0.29 ± 0.0014 a	0.31 ± 0.0077 c	2	7%	−7%
pH = 7.0	0.09 ± 0.0013 c	0.53 ± 0.0197 a	2	494%	295%
pH = 9.0	0.11 ± 0.0059 b	0.37 ± 0.0278 b	2	231%	153%
EC					
pH = 3.5	0.09 ± 0.0011 a	0.23 ± 0.0036 b	2	204%	167%
pH = 7.0	0.05 ± 0.0008 b	0.35 ± 0.0095 a	2	680%	603%
pH = 9.0	0.10 ± 0.0016 a	0.51 ± 0.0232 c	1	432%	218%
CA					
pH = 3.5	0.13 ± 0.0005 a	0.35 ± 0.0042 b	2	168%	154%
pH = 7.0	0.05 ± 0.0016 c	0.61 ± 0.0087 a	5	1041%	929%
pH = 9.0	0.10 ± 0.0006 b	0.32 ± 0.0093 c	3	233%	139%
CGA					
pH = 3.5	0.07 ± 0.0022 b	0.22 ± 0.0207 a	1	230%	186%
pH = 7.0	0.05 ± 0.0012 c	0.39 ± 0.0133 c	2	673%	616%
pH = 9.0	0.09 ± 0.0017 a	0.19 ± 0.0042 b	2	117%	90%

Values expressed are mean ± standard deviation. Values within the same row with different letters are significantly different at *p* < 0.05.

**Table 2:** The affinity of four compounds and eugenol with tyrosinase and the amino acid residues involved in hydrophobic interactions and hydrogen bonds with four compounds.

Ligands	Affinity (kJ mol^−^^1^)	Number of Hydrophobic Interactions	Amino Acid Residues Involved in Hydrophobic Interactions	Number of Hydrogen Bonds	Amino acid Residues Involved in Hydrogen Bonding
CT	−7.0	7	His61, His85, His263, Phe264, Ser282, Val283, Ala286	3	His244, Asn260, Met280
EC	−6.7	8	His61, Val248, His263, Phe264, Asn260, Val283, Ser282, Ala286	2	Met280CuB
CA	−6.3	8	Val248, Asn260, His263, Phe264, Gly281, Ser282, Val283, Ala286	2	His244, Met280
CGA	−6.9	8	His61, Asn260, His263, Phe264, Pro277, Ser282 Val283, Ala286	3	Gly281, Arg268, Met280
Eugenol	−6.1	8	Gly280, Asn260, Phe264, His259, His263, His85, Val283, Ser282	1	Met280

Eugenol is located at the hydrophobic pocket, surrounded by the residues Gly280, Asn260, Phe264, His259, His263, His85, Val283, Ser282, and Met280, forming a strong hydrophobic binding (Figure 14i,j). Detailed analysis showed that the phenyl group of the eugenol formed π-π interaction with the residue Met280.

## Data Availability

Data is contained within the article.
